# Cleaner and Sustainable Production of Core–Sheath Polymer Fibres

**DOI:** 10.3390/polym16162357

**Published:** 2024-08-20

**Authors:** Manul Amarakoon, Anthony Harker, Shervanthi Homer-Vanniasinkam, Mohan Edirisinghe

**Affiliations:** 1 Department of Mechanical Engineering, University College London, Roberts Engineering Building, Torrington Place, London WC1E 7JE, UK; manul.amarakoon.21@ucl.ac.uk (M.A.); s.homer-v@ucl.ac.uk (S.H.-V.); 2 Department of Physics and Astronomy, University College London, Gower Street, London WC1E 6BT, UK; a.harker@ucl.ac.uk

**Keywords:** energy, sustainability, polymer, fibre, production

## Abstract

The amalgamation of sustainable practises throughout the fabrication process with advanced material engineering holds promise not only for eco-conscious manufacturing but also for promoting technological advancements in versatile material design and application. Moreover, technological innovation serves as a catalyst for sustainability initiatives, driving innovation and enabling the adoption of greener practises across industries. This study investigates redefining the production protocol of pressure spinning to produce core–sheath polymer fibres, deepening sustainable practises. It aims to explore innovative approaches such as modifying spinning parameters, optimising polymer solvent configurations and understanding fluid behaviour to curtail material wastage and maintain minimal energy consumption without compromising production efficiency. Utilising Polyvinylpyrrolidone (PVP) for the core and Polyethylene oxide (PEO) for the sheath, production rates of up to 64 g/h were achieved with a fibre diameter range of 3.2 ± 1.7 µm to 4.6 ± 2.0 µm. Energy consumption per mass of fibres produced showed a decreasing trend overall with increasing applied gas pressure. These findings highlight the potential for the efficient and scalable production of core–sheath fibres with applications in various advanced materials fields.

## 1. Introduction

The pursuit of sustainable practises within the domain of material synthesis and manufacturing methods stands as a cornerstone in modern scientific endeavours. In the area of polymeric fibre production, the evolution of core–sheath fibres offers a paradigm shift towards multifunctional materials with diverse applications [1]. The core–sheath structure of polymer fibres refers to forms of fibre where a ‘core’ polymeric fibre material is coated using another polymeric material, forming the ‘sheath’ of the overall fibre. Depending on the application, the advantages of this fibre structure include enhancing the mechanical properties of the overall fibre, improving electrical conductivity in electronic applications and revamping electrochemical performance for efficient energy storage [2,3,4,5]. These fibres are also used in filtration; for instance, the reusability and longevity of core–sheath structures are the subjects for research in the field of water treatment [6].

Core–sheath fibres are often used in scaffolds and drug-delivery systems [7,8,9]. A typical case is when the core can contain drugs or therapeutic agents, while the sheath provides a protective barrier. The core–sheath design also enables controlled release kinetics, enhancing the efficacy and duration of the therapeutic effect [10,11]. Furthermore, the structure allows for biocompatibility where the use of biocompatible materials in the core–sheath fibre design ensures compatibility with biological systems [12]. This is essential for applications such as tissue engineering and implantable medical devices [13].

However, the traditional techniques utilised in the fabrication of these fibres often entail inefficiencies, leading to material wastage and high-power consumption [14]. Core–sheath polymeric fibres in the micrometre and nanometre scale are produced using multiple methods where each has its advantages along with limitations. The methods include template assisted methods, layer-by-layer assembly, solution blowing, gyration-based methods and electrospinning methods such as coaxial electrospinning and emulsion electrospinning [2,15,16,17,18,19]. There has been a strong focus in the literature on producing core–sheath fibres based on the specific application requirements. However, the sustainability of producing these fibres while considering the production efficiency and energy consumption is understudied.

The United Nations Environment Assembly has prioritised the establishment of a globally binding agreement on plastic pollution [8]. To realise this objective, it is essential to advance sustainable methods in the production of polymer-based components, including fibres. An effective method of improving sustainability in the production of the polymeric fibres is the consideration of the 12 Principles of Green Engineering and the 12 Principles of Green Chemistry [20,21]. These principles align with broader goals of sustainability and responsible resource management. Arguably, the two frameworks emphasise three key pillars: energy efficiency, the use of non-hazardous materials and minimal wastage. Incorporating these elements in polymeric fibre production will develop processes and polymer products that are not only environmentally friendly, but also economically viable and socially responsible.

Core–sheath pressure spinning (also called pressurised gyration) is a relatively simple and efficient process where the extrusion of fibre is supplemented by centrifugal force coupled with gas pressure [17]. This gyration-based method is more efficient than methods that produce polymeric nanofibres when both the production rate and energy consumption are considered [14]. However, this method can be further optimised for improved production efficiency and energy consumption. The operation of producing core–sheath fibres via pressurised gyration involves rapidly extruding polymer solutions through a spinneret that consists of two reservoirs to hold the polymeric solutions of the core and the sheath of the fibres produced through co-axial orifices in the outer wall of the spinneret.

The spinning process of core–sheath pressurised gyration can use excess energy to produce fibres if the required process control parameters are ill-understood. Forming fibres from polymer solutions using this method can require high rotary speeds, applied pressure magnitudes or changes to the environment such as temperature and humidity control, where achieving these parameters is typically associated with increasing power consumption. Attaining specific temperatures and humidity control typically requires energy-intensive heating or cooling systems, contributing to elevated power consumption. However, process optimisation with a focus on varying solution characteristics rather than varying other parameters can augment energy efficiency in pressurised gyration.

The time required to spin fibres from a specific quantity of polymeric solution is directly proportional to the energy consumption of the forming of fibres with pressurised gyration, where the spin time of polymeric solutions depends on the process control parameters [22]. Process control parameters dictate how fast a polymer solution is extruded into fibres or if there will be any fibres fabricated at all. The time to produce fibres is also critical in achieving a high production rate to enhance the overall efficiency and sustainability.

**Figure 1:**
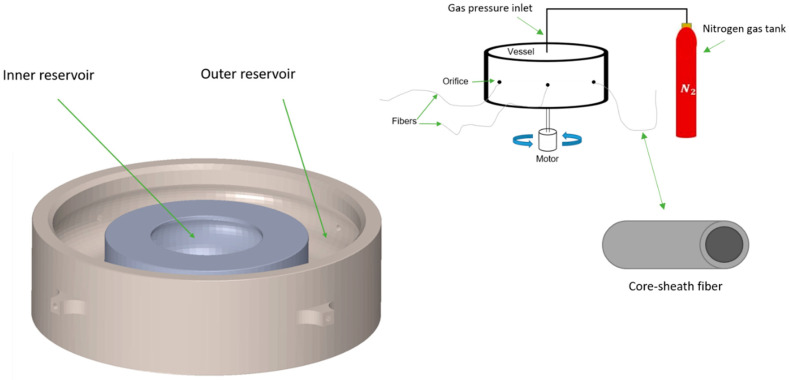
Schematic of the core–sheath pressurised gyration method (**right**), along with an internal view of the core–sheath vessel (**left**).

To enhance sustainability by maintaining minimal material wastage in core–sheath pressurised gyration, there is a need to ensure all fibres formed have a core–sheath structure. Hence, it is pivotal to understand the fluid dynamics within the coaxial orifices that jet out core–sheath fibres. The cross-section of the orifices of the vessel shows that the solution used for the core travels through a tube, whereas the solution to form the sheath moves through an annulus to jet out core–sheath fibres out of the vessel (Figure 1). Assessing the pressure difference at both ends of the core tube and sheath tube allows for the determination of the volumetric flow rates using Poiseuille’s Law [23].

Through a systematic evaluation of sustainable methodologies and their implications on fibre quality, this paper seeks to illuminate pathways for a greener and more efficient production landscape in the domain of core–sheath fibre synthesis. The study highlights how this can be carried out to produce core–sheath fibres with a distinct dual-phase composition using Polyethylene oxide (PEO) and Polyvinylpyrrolidone (PVP). The choice of PEO and PVP as the core–sheath fibre materials is informed by previous research experience, where their compatibility and advantageous properties have been extensively studied and documented in the literature [22]. PVP is non-toxic and water-soluble, whilst PEO has a very low single-dose oral toxicity. Therefore, the two polymers abide by the Principles of Green Chemistry [24,25].

## 2. Material and Methods

### 2.1. Materials

Polyethylene Oxide (PEO) (Mw = 200,000 g mol^−1^), Polyvinylpyrrolidone (PVP) (Mw = 1,300,000 g mol^−1^) and Rhodamine B were obtained from Sigma Aldrich (Gillingham, UK). Deionised water was used as a solvent for both PEO and PVP.

### 2.2. Solution Preparation

The solution for the core of the fibres used two concentrations of PVP dissolved in deionised water at concentrations of 50 wt.% and 60 wt.%. The solutions for the sheath of the fibres involved two concentrations of PEO 40 wt.% and 50 wt.%, along with Rhodamine 1 wt.% dissolved in deionised water. All solutions were magnetically stirred for 24 h to ensure homogeneity. The solution viscosities used in this study were characterised using a calibrated Brookfield viscosity-meter (AMETEK Brookfield, Harlow, UK) along with a LV-4 (64) spindle attachment. The viscosities of the PEO solutions with PEO 40% and PEO 50% both mixed with Rhodamine 1% were evaluated to be 146 Pa s and 405 Pa s, respectively, and PVP 50% and PVP 60% were shown to have viscosities of 124 Pa s and 240 Pa s. To obtain a valid measurement, the torque value of the rotational viscometer used in this study must be between 10% and 100%, where higher torque values indicate better accuracy [26]. Hence, the above viscosity readings were captured at 100% torque for the best accuracy.

In previous work, the water-soluble polymers, PEO and PVP, were shown to effectively produce fibres via pressurised gyration at the selected concentrations [22]. Specifically, these polymers demonstrated excellent solubility in the chosen solvent (deionised water), appropriate viscosity levels, and compatibility with the process parameters, thus enabling the successful production of fibres. Hence, based on experience and the formability of the fibres, along with the range of the magnitudes of process control parameters that were available, PEO was selected for the sheath and PVP was selected for the core. PEO was deemed suitable for the sheath component, due to its excellent film-forming properties which contribute to the functionality of the resulting core–sheath fibres [27]. Conversely, PVP, recognised for its mechanical stability, was selected for the core to enhance structural integrity [28].

### 2.3. Core–Sheath Pressurised Gyration

Figure 1 depicts the experimental setup along with a closer internal look at the open rotary vessel that shows the inner reservoir and the outer reservoir that hold the solution to produce the core and the sheath, respectively. The largest internal diameter of the inner reservoir (which is on the same plane as the connecting tubes to the orifices) is 36 mm. The largest internal diameter of the outer reservoir is 70 mm, whereas the diameter of the external wall of the vessel up to the end of the orifices from opposing ends is 80 mm. The system includes a pressurised nitrogen gas inlet connected to the top of the vessel, controlled using a pressure gauge that can deliver up to 0.3 MPa of applied gas pressure into the vessel. The fibres produced for characterisation were sampled from the wall of the collector, which is 150 mm away from the external wall of the vessel. 

The rotary vessel used in this study consists of 4 co-axial orifices. The connecting tube that moves the solution from the inner reservoir through the orifice to form the core of fibre is reinforced at the external wall of the inner reservoir with fillets. Similar fillets are shown in Figure 1 in the outer wall of the vessel where the orifices protrude 3 mm from the outer wall of the vessel. This allows the annulated tube of the orifice which forms the sheath of the fibres to be of 5 mm in length. Previous work has shown that the incorporation of a deeper orifice stabilises the flow state of the solution and better promotes the formation of polymer jets parallel to the axis of the tubes of the orifice [29]. This is paramount in the design of the core–sheath vessels, as the sheath travels in a tube that is significantly shorter than the tube which forms the core. Hence, the addition of protruding orifices allows for a better formation of both the core and the sheath polymer jets. Furthermore, the incorporation of fillets to the design maintains the concentricity of the orifice. It is important for the tubes in the orifice to have a common centre to support a uniform width of the sheath around the core of the fibres produced.

The vessel also uses fillets in the internal walls of both the outer and inner reservoirs. The fluid behaviour within the reservoir of the vessel when spun is known to have a parabolic shape as it reaches the tubes in the inner walls of the gyration-based fibre-manufacturing vessels [17]. Therefore, the fillets within the internal walls of the reservoirs support the movement of the solution and their necessity is further exemplified when dealing with a high viscous polymer solution, as in this study.

### 2.4. Fibre Production

Core–sheath fibres were generated by loading both reservoirs with 2 mL of solution; in all experiments, spinning was carried out for 30 s to maintain consistency throughout the analysis. However, for each core–sheath polymer configuration, the spinning process was commenced with the extrusion of PVP solution loaded in the core, leaving the outer reservoir for the sheath empty to produce PVP fibres. The process was then repeated by loading both the PVP solution in the core and the PEO solution mixed with Rhodamine 1% in the sheath, leading to the formation of core–sheath fibres characterised by a visible, pink-dyed sheath surrounding an undyed PVP core. Hence, two samples were obtained at the same magnitudes of the forming parameters, where the first is ‘core-only’ PVP fibres and the second sample is core–sheath fibres. The mass of fibre samples was measured using a microscale and the production rates were evaluated by dividing the mass of the samples by the spin time. The difference in the two production rates of the samples was used to evaluate the production rate of the sheath in the core–sheath sample.

### 2.5. Evaluation of Process Parameters

The Nichibo DC motor connected to the vessel has an unloaded power rating of 21.2 W, which was seen to draw a power of 28 W when it was run with the vessel attached. The power increased to 28.7 W, 30.7 W and 33.7 W at applied gas pressure magnitudes of 0.1 MPa, 0.2 MPa and 0.3 MPa, respectively. The power readings were captured using a Maxcio Energy Monitor (Shenzhen, China) which measured real-time power drawn by the motor, whereas the rotational speed of the vessel was measured using a laser tachometer.

### 2.6. Fibre Characterisation

**Figure 2:**
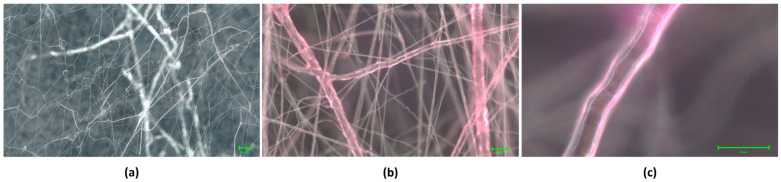
(**a**) Core-only PVP fibres. (**b**) Core–sheath PEO-PVP fibres. (**c**) Close-up of core–sheath fibre.

Optical microscopy was used to confirm the formation of a sheath around the core that resulted in core–sheath fibres, as shown in Figure 2. The overall fibre dimensions were evaluated using a Scanning Electron Microscope (GeminiSEM 360, Carl Zeiss Microscopy GmbH, Oberkochen, Germany) The difference in the fibre diameter of the two samples (core-only and core–sheath) was used to evaluate the width (or radius) of the sheath of the resulting core–sheath fibres. Fibre dimensions were calculated using Image J (version 1.49) analysis where 100 randomly selected fibres from Scanning Electron Microscope (SEM) images were averaged.

To further confirm the presence of PEO and PVP when producing core–sheath fibres, Fourier Transform Infrared Spectroscopy (FTIR) was undertaken using a Thermo Fisher Scientific, Nicolet iS50 FTIR (Thermo Scientifc, Waltham, MA, USA). In total, 2 mg of the PEO, PVP and core–sheath fibre samples was placed on the ATR crystal and evaluated over 10 rounds in the range of 4000–1000 cm^−1^ at a resolution of 4 cm^−1^ to record the measurements.

### 2.7. Analytical Modelling

**Figure 3:**
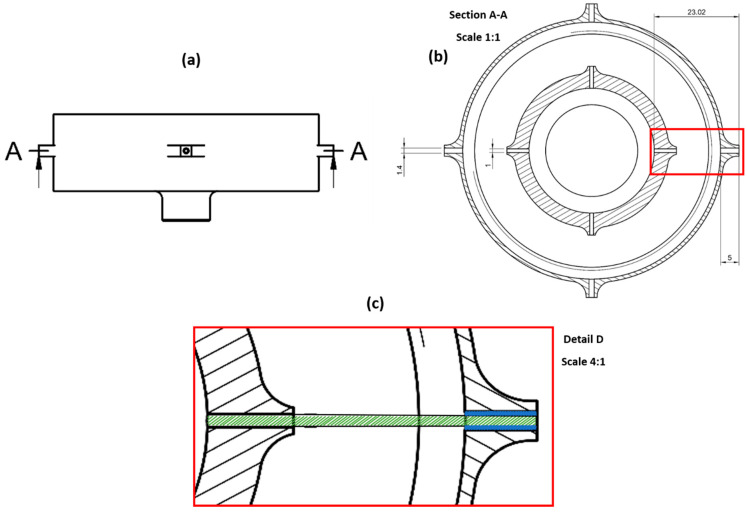
(**a**) External view of the vessel. (**b**) Cross-sectional view of section A-A. (**c**) Close-up view of detail D, portraying the simple tube (green) and annular tube (blue) connecting the inner reservoir and outer reservoir, respectively, to the external wall of the vessel.

The volume flow rates of the polymer solutions used in this study were evaluated by considering the dimensions of the vessel, the magnitude of rotary speed, fluid viscosities and pressure differences. The dimensions of the vessel are shown in Figure 3, where a close-up cross-sectional schematic of the vessel is included (within the red box). It is assumed that flow within the coaxial system of the vessel is laminar, considering the relatively higher viscosities of the polymeric solutions used in this study [30].

Using the dimensions of the simple tube of radius r (r = 0.34 mm) that connects the inner reservoir to the orifice, the volumetric flow rate of the polymeric solution used to produce the core of the core–sheath fibres formed is calculated as follows [31]:

$$Q_{core}=\frac{\pi \left(\Delta P\right)r^{4}}{8\times pipe\;length\times viscosity}$$

The volumetric flow rate of the solution to produce the sheath is calculated as follows, considering the solution moves through an annular tube (indicated in Figure 3) to reach the orifice. If $r_{2}$ and $r_{1}$ are the external radius (0.7 mm) and internal radius (0.5 mm) of the annular tube:

$$Q_{sheath}=\frac{-\pi \left({r_{2}}^{2}-{r_{1}}^{2}\right)}{8\times viscosity}\left(\left({r_{2}}^{2}-{r_{1}}^{2}\right)-\frac{\left({r_{2}}^{2}-{r_{1}}^{2}\right)}{\ln\left(\frac{r_{2}}{r_{1}}\right)}\right)\frac{\Delta P}{annular\;tube\;length}$$

The effective pressure difference in both the simple tube and the annular tube is calculated as follows:

$$\Delta P=P_{1}+\frac{1}{2}\rho \omega^{2}\left(R^{2}-{R_{0}}^{2}\right)$$

‘$P_{1}$’ is the magnitude of applied gas pressure, ‘R’ is the distance from the centre of the vessel to the end of the orifice (tube length), ‘$R_{0}$’ is the inner radius of the reservoir, ‘ρ’ is the density of the fluid and ‘ω’ is the angular velocity.

## 3. Results and Discussion

The overall experimental results are categorised according to the configuration of the core–sheath configurations as shown in Table 1, Table 2, Table 3 and Table 4. The presence of core–sheath fibres was not evident when the gas pressure was not applied using a core–sheath configuration of PEO 40% in the sheath, indicated as ‘no core–sheath (C-S)’ in Table 1 and Table 2. However, the formation of core–sheath fibres with PEO 40% was seen when pressure was introduced, and it was seen to further improve when the magnitude of the applied pressure was increased. Regardless, not all of the produced fibres were core–sheath, and hence, the core–sheath results for fibre mass and production rates were ignored, as indicated as ‘not all c-s’ in Table 1 and Table 2. Therefore, the mass estimates along with the production rate estimates for the core–sheath fibres that used PEO 40% in the sheath were not considered. The core–sheath fibre dimensions when using PEO 40% were evaluated by distinguishing the fibres that showed a pink dye coating in the optical images of samples, as a pink coating demonstrates that the fibre is core–sheath. The area of samples produced using PEO 40% that showed a full sheath under optical microscopy was analysed using SEM to obtain the fibre dimensions.

**Figure 4:**
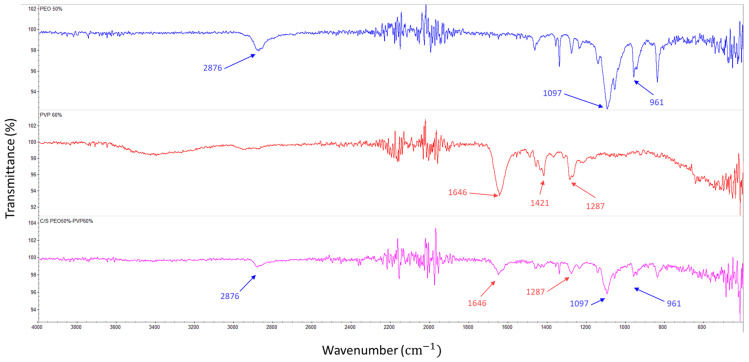
FTIR spectra PEO fibres, PVP fibres and core–sheath PEO50%–PVP60% fibres.

FTIR spectrum for PEO fibres show an absorption at 2875.64 cm^−1^ corresponding to the molecular stretching of the methylene group CH_2_, whereas the peaks at 1097.01 and 961.32 cm^−1^ are caused by the stretching of the ether group in PEO which is further indicated as the C–O–C absorption complex [32]. The spectrum for PVP indicated a peak at 1646.01 cm^−1^ which proved the stretching of C-O, whilst the C-H bending and CH_2_ wagging were observed at 1420.79 cm^−1^ and 1287.19 cm^−1^, respectively [1]. The presence of these peaks in the core–sheath sample is evident and this corresponds to the presence of both PEO and PVP as shown in Figure 4.

Applying the theory of volumetric flow rate, the optimum parameters to form core–sheath fibres with minimal material wastage, using the same volume of solution in both the inner and outer reservoir of the vessel, was calculated to be PEO 40% in the sheath and PVP 60% in the core with no applied gas pressure. Minimal wastage in this case means that once both solutions travel through the orifice at the same time. For instance, if the time taken for a specific quantity of solution to move from the outer reservoirs to form the sheath is half of the time taken for same quantity of solution to move from the inner reservoir to form the core, this would mean that only half of the fibres produced are core–sheath. The time taken for the volume of solution in the two reservoirs to eject through (either the simple tube or annulated tube depending on core or sheath) to the orifices was calculated based on the number of orifices and flow rates through either tube.

$$Time=\left(\frac{Volume\;in\;reservoir}{Number\;of\;orifices}\right)÷Flow\;rate$$

The theoretical time to spin identical volumes of PVP 60% in the core and PE0 40% in the sheath results in the best ‘core–sheath’ volumetric flow rate ratio that is closest to a ratio of 1:1.

When considering fibre quality, production rate and energy consumption, the experimental validation of the polymer configurations used in this study, the best core–sheath fibres resulted when PVP 60% is used in the core and PEO 50% is used in the sheath (see Section 3.2). For the PVP 60% by PEO 50% core–sheath configuration, the theory of volumetric flow rate shows that the most desirable ‘core–sheath’ volumetric flow rate ratio is achieved at an applied pressure magnitude of 0.3 MPa, which resulted in a volumetric flow rate ratio of 0.52. This is considering the magnitudes of process control parameters available and when the same volumes of solutions are used in the core and the sheath. To maintain minimal material wastage, it is rational to utilise the theory of volumetric flow rate to estimate the amounts of polymeric solution to use in the inner reservoir and the outer reservoir, once the optimum core–sheath fibre forming parameters are experimentally validated. Hence, using a 2:1 core to sheath volume ratio to form core–sheath fibres using PVP 60% and PEO 50% at an applied pressure magnitude of 0.3 MPa will equate to a core to sheath volumetric flow rate ratio of 1.06.

### 3.1. Production Rate

**Figure 5:**
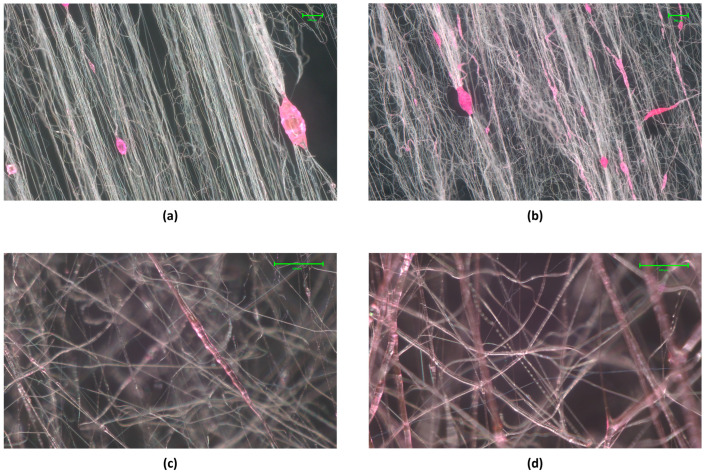
Optical images of attempt to produce core–sheath fibre using PEO 40% in the sheath at applied pressure magnitudes of (**a**) 0 MPa, (**b**) 0.1 MPa, (**c**) 0.2 MPa and (**d**) 0.3 MPa.

The results obtained when using PEO 40% as the sheath were ignored in the production rate analysis as it is not feasible to separate the fibres that are core–sheath from the fibres that are not. Nevertheless, optical microscopy proved that the application of pressure improved the forming of core–sheath fibres using PEO 40% in the sheath, as seen in Figure 5.

**Figure 6:**
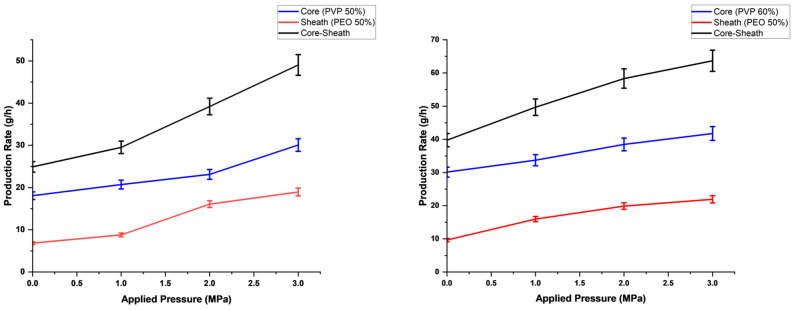
Production rate against applied pressure magnitudes.

The production rates increased with the application of pressure for all configurations of the core–sheath fibre samples. This is seen in the core and in the sheath of the fibre separately and in the fibre as a whole (Figure 6). The production rate also has an increasing trend as polymer concentration is increased. This shows that although the viscosity is paramount in terms of the volumetric flow rate through the orifices of the vessel, it does not necessarily mean this reflects on the actual fibres produced, as the resulting solid fibres that are studied are not of liquid state. In theory, the volumetric flow rate of a solution is inversely proportional to it viscosity. However, in this study, the production rate is a measure of the mass of fibres formed per unit time, rather than a measure of the mass of solution jetted through the orifices.

### 3.2. Energy

The necessity of applied pressure magnitudes to produce core–sheath fibres were signified when using PEO 40% in the sheath. However, the increasing addition of applied pressure magnitudes result in the increase in power consumption by the motor, along with a slight decrease in the rotary speed of the vessel. The decrease in rotary speed at specific magnitudes of applied gas pressures were taken into consideration in the volumetric flow rate analysis. The change in rotary speed of the spinning vessel with the addition of gas pressure is attributed to the need for more torque to maintain rotational speed caused by the additional load on the motor. In a DC motor, the output torque is directly proportional to the current. Hence, an increase in power drawn is witnessed, as power is the product of voltage and current.

**Figure 7:**
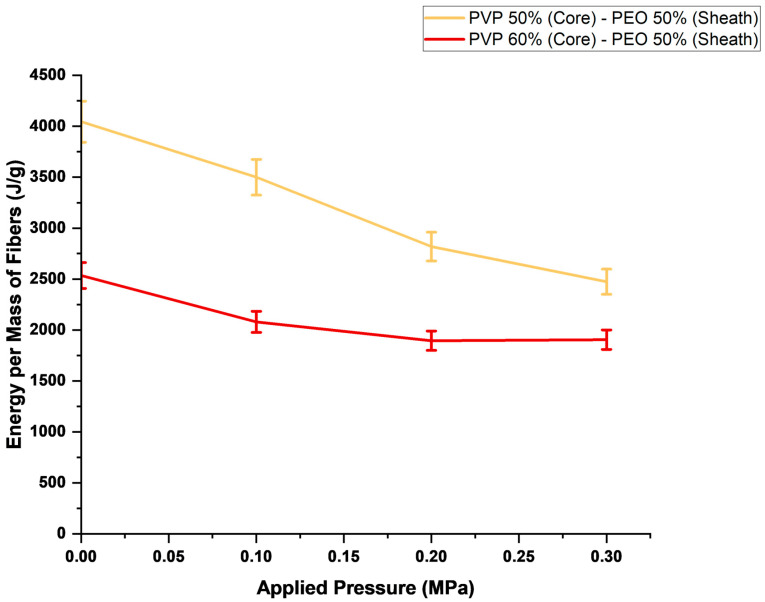
Energy consumption per mass of fibres produced.

Figure 7 depicts the energy per mass of a core–sheath fibre produced at applied pressure magnitudes. The graph depicts only the forming of fibres using PEO 50% in the sheath, as not all fibres formed using PEO 40% in the sheath were characterised as fully core–sheath. A predominantly decreasing energy consumption is seen with the application of higher-pressure magnitudes to produce fibres. It should be noted that the energy usage associated with the pressurisation of the gas is not included, as this gas pressurisation is pre-processed. The pressurised gyration process involves the release of gas from a tank to obtain the desired applied gas pressures. Hence, there is no energy requirement to apply gas pressure magnitudes during fibre production.

Figure 7 shows that using PVP 60% over PVP 50% for the core of the fibres results in a more energy-efficient production with respect to the mass of fibres produced. However, it is seen that when using PVP 60% in the core at an applied pressure magnitude of 0.3 MPa, the energy required to produce a specific mass of fibres is higher than that to produce the same mass of fibres with PVP 60% at 0.2 MPa, due to the increase in power consumption due to higher applied pressure magnitudes. Regardless, increasing applied pressure magnitudes is shown to improve processing energy efficiency, and the application of pressure to produce sustainable fibres in a gyration-based process is exemplified.

### 3.3. Fibre Dimensions

**Figure 8:**
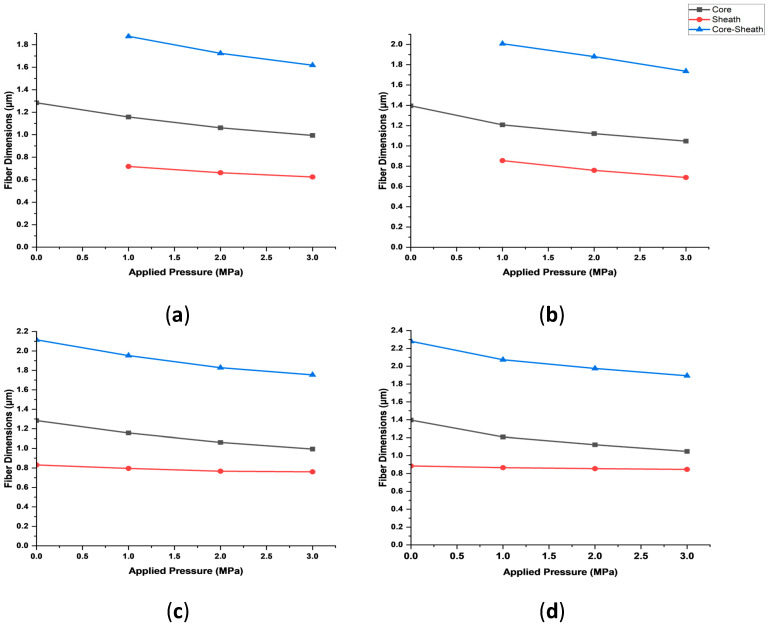
Fibre dimensions against applied pressure magnitudes. (**a**) PVP 50% (core)—PEO 40% (sheath). (**b**) PVP 60% (core)—PEO 40% (sheath). (**c**) PVP 50% (core)—PEO 50% (sheath). (**d**) PVP 60% (core)—PEO 50% (sheath). The standard deviation of the results is shown in Table 1, Table 2, Table 3 and Table 4.

Under the same magnitudes of affecting parameters, the diameter of the core-only fibre is likely to be smaller than the core of the core–sheath configuration, as the core of the core–sheath fibre is not exposed to the atmosphere during jetting. Hence, the core of a core–sheath fibre experiences less solvent evaporation in comparison to a core-only fibre. Regardless, the evaluation of the fibre dimensions assumes that the diameter of the core in the core–sheath configuration will be the same as in the core-only fibre. Figure 8 depicts the effects of applied pressure magnitudes on fibre dimensions that includes the radius of the core, the thickness of the sheath and the radius of the overall core–sheath fibres.

The decrease in cross-sectional dimensions under increasing pressure magnitudes associated with polymeric fibres that are produced via pressurised gyration is evident. An increase in concentration of the solutions is also shown to increase the overall diameter of the core–sheath fibres produced. This is attributed to the higher quantity of polymers that is in the polymeric solution of higher concentrations. Hence, when the solvent is evaporated, the polymeric fibres that are formed are larger.

Figure 8 shows that for a specific configuration, the radius of the core decreases with the increase in applied pressure magnitudes. However, the width of the sheath seems to exhibit a lower decreasing trend in comparison to the radius of the core with the application of pressure. Moreover, using PEO 50% in the sheath shows that the width of the sheath remains almost the same with the application of increasing pressure magnitudes. This is attributed to the effective pressure difference which drives the solution through the inner tube of the orifice to the core being 2.4 (at 0.3 MPa applied pressure) to 3.5 times (at no applied pressure) larger than the pressure difference created along the comparatively shorter annular tube that produces the sheath. Therefore, the core of the fibres produced will experience more driving force in comparison to the sheath, which promotes more elongation in the core in comparison to the sheath. This, along with the difference in the solution characteristics where PEO 50% has a higher viscosity than the PVP solutions, justifies the fibre dimensions obtained, as the lower viscous PVP solutions are easier to elongate than the higher viscous PEO 50% solution.

**Figure 9:**
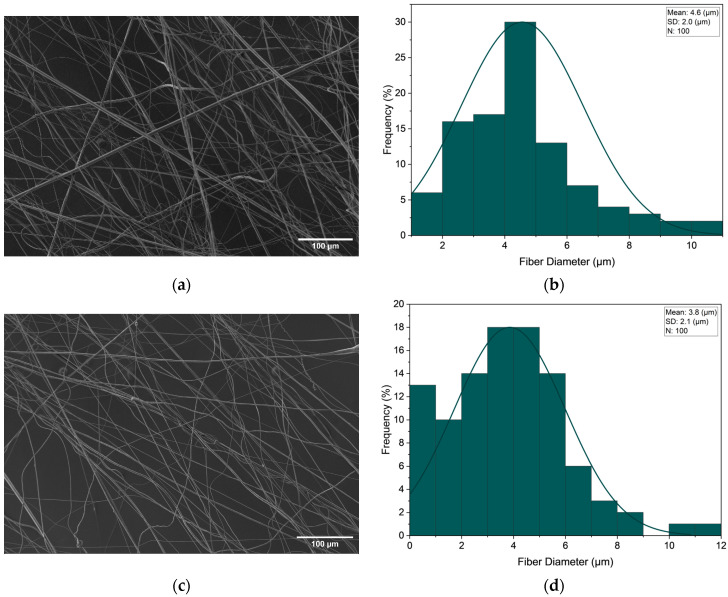
SEM image at no applied pressure (**a**) along with fibre distribution (**b**) and SEM image at 0.3 MPa applied pressure (**c**) along with fibre distribution (**d**) for PVP 60% and PEO 50% core–sheath fibre configuration.

In all core–sheath polymer solution configurations, a decrease in fibre diameter along with an increase in variance was seen with the application of increasing pressure magnitudes. Figure 9 portrays the scanning electron micrographs along with the fibre diameters distributions for the PVP 60% and PEO 50% core–sheath configuration at the minimum and maximum applied pressure magnitudes (no applied pressure and 0.3 MPa).

**Figure 10:**
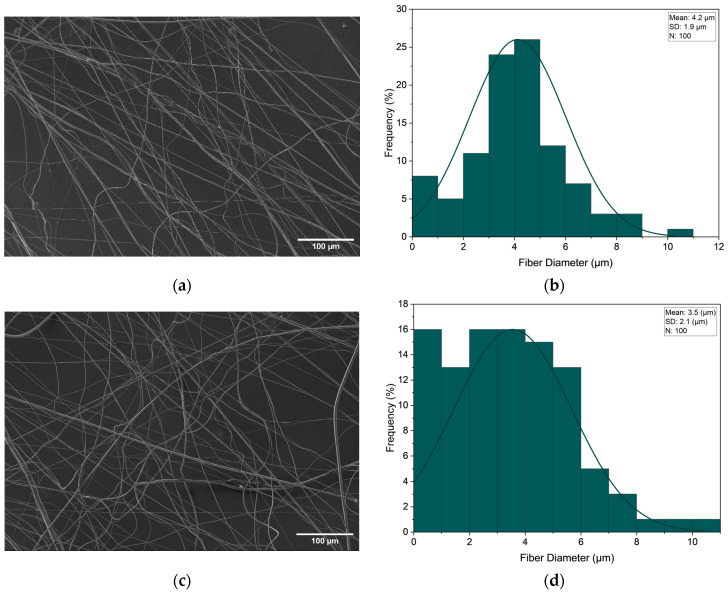
SEM image at no applied pressure (**a**) along with fibre distribution (**b**), and SEM image at 0.3 MPa applied pressure (**c**) along with fibre distribution (**d**) for PVP 50% and PEO 50% core–sheath fibre configuration.

In comparison to the PVP 60% by PEO 50% core–sheath configuration, the PVP 50% by PEO 50% configuration shown in Figure 10 shows that the fibre diameters are smaller at the same magnitudes of applied pressure. Both configurations show an increase in variance with applied gas pressure as the standard deviation increases with the application of gas pressure. However, this increase in standard deviation associated with the application of pressure is not very significant in comparison to previous studies that used lesser viscous polymer solutions to produce fibres via pressurised gyration [29,33]. Regardless, a higher viscosity serves to enhance flow stability, as solutions exhibit greater resistance to deformation and flow fluctuations. This increased resistance makes them less susceptible to jet instabilities, ultimately leading to the production of more consistently sized fibres. Hence, in the instance where fibre uniformity is significant, using higher viscous polymeric solutions is a more sustainable approach.

In comparison to water-soluble polymers, non-water-soluble polymers are less sensitive to variations in relative humidity [34,35]. This is attributed to the higher interaction of water-soluble polymers with moisture in the air, leading to changes in their physical properties and processing behaviour [36]. In comparison, non-water-soluble polymers are less affected by humidity fluctuations. Regardless, water-soluble polymers are preferred as they abide by the Principles of Green Chemistry, as they do not require hazardous solvents.

Optimising the polymer concentration of non-water-soluble polymer solvents can mitigate the impact of environmental conditions as a higher concentration promotes solidification. This study showed that higher polymer concentrations provide better control over the fibre formation process and the sensitivity to environmental conditions. Achieving a sufficient viscosity when using non-water-soluble polymers allowed for effective fibre production, without the need for strict control over temperature and humidity. Overall, this can be advantageous in practical applications, where maintaining precise environmental conditions can be both economically and environmentally challenging.

The limitations of this study such as the use of Pouseillie’s Law to determine the flow rates of the solution based on effective pressure difference assumes that the polymer solution used in this study is incompressible and the flow is laminar. Furthermore, it is also acknowledged that the uncertainty associated with the width of the sheath may be large, considering it is calculated by evaluating the difference in the mean and standard deviations of the core and the core–sheath fibre dimensions. Regardless, this study provides a useful approximation, where despite the challenges, the insights gained from this study contribute to our understanding of the sustainable production of fibres via pressurised gyration.

## 4. Conclusions

This investigation into core–sheath fibre production via pressurised gyration unravels the intricate relationship between process control parameters, fluid dynamics and resultant fibre dimensions, along with energy and production efficiency. Core–sheath fibres produced using higher concentrations of polymer solution under higher applied pressures were more energy and production efficient. Empirical validation considering all process control parameters proved to be more effective than relying solely on theoretical volumetric flow rate calculations, guiding the selection of optimal polymer concentrations for tailored core–sheath fibre production. This study shows how varying pressure magnitudes and polymer concentrations influence fibre dimensions and uniformity, with higher viscosity polymer solutions yielding more consistently sized fibres, whilst mitigating the need for strict control over temperature and humidity. Despite inherent limitations, our findings contribute valuable insights into sustainable fibre production methods. Moving forward, further research can explore how other parameters, such as the collector distance, may influence fibre production sustainability.

## Figures and Tables

**Table 1:** Experimental results of core–sheath configuration of PVP 50% and PEO 40%.

	PVP 50/PEO 40							
	Core	C-S	Core	C-S	Core	C-S	Core	C-S
Pressure (MPa)	0		0.1		0.2		0.3	
Speed (RPM)	12,000		12,000		11,800		11,600	
Fibre mass (g)	0.15 ± 0.01	no c-s	0.17 ± 0.01	not all c-s	0.19 ± 0.01	not all c-s	0.25 ± 0.01	not all c-s
Production rate (g/h)	18 ± 0.9	no c-s	21 ± 1.0	not all c-s	23 ± 1.2	not all c-s	30 ± 1.5	not all c-s
Diameter (µm)	2.6 ± 1.2	no c-s	2.3 ± 1.2	3.8 ± 1.8	2.1 ± 1.1	3.4 ± 1.8	2.0 ± 1.0	3.2 ± 1.7
Sheath thickness (µm)	No sheath formed		0.8 ± 1.1		0.7 ± 1.1		0.6 ± 1.0	

**Table 2:** Experimental results of core–sheath configuration of PVP 60% and PEO 40%.

	PVP 60/PEO 40							
	Core	C-S	Core	C-S	Core	C-S	Core	C-S
Pressure (MPa)	0		0.1		0.2		0.3	
Speed (RPM)	12,000		12,000		11,800		11,600	
Fibre mass (g)	0.25 ± 0.01	no c-s	0.28 ± 0.01	not all c-s	0.32 ± 0.02	not all c-s	0.35 ± 0.02	not all c-s
Production rate (g/h)	30 ± 1.5	no c-s	34 ± 1.7	not all c-s	39 ± 2.0	not all c-s	42 ± 2.1	not all c-s
Diameter (µm)	2.8 ± 1.3	no c-s	2.4 ± 1.3	4.0 ± 1.9	2.2 ± 1.2	3.8 ± 1.9	2.1 ± 1.2	3.5 ± 1.8
Sheath thickness (µm)	No sheath formed		0.8 ± 1.2		0.8 ± 1.1		0.7 ± 1.1	

**Table 3:** Experimental results of core–sheath configuration of PVP 50% and PEO 50%.

	PVP 50/PEO 50							
	Core	C-S	Core	C-S	Core	C-S	Core	C-S
Pressure (MPa)	0		0.1		0.2		0.3	
Speed (RPM)	12,000		12,000		11,800		11,600	
Fibre mass (g)	0.15 ± 0.01	0.21 ± 0.01	0.17 ± 0.01	0.25 ± 0.01	0.19 ± 0.01	0.32 ± 0.02	0.25 ± 0.01	0.41 ± 0.02
Production rate (g/h)	18 ± 0.9	25 ± 1.3	21 ± 1.0	30 ± 1.5	23 ± 1.2	39 ± 2.0	30 ± 1.5	49 ± 2.45
Diameter (µm)	2.6 ± 1.2	4.2 ± 1.9	2.3 ± 1.2	3.9 ± 1.9	2.1 ± 1.1	3.7 ± 2.0	2.0 ± 1.0	3.5 ± 2.1
Sheath thickness (µm)	0.8 ± 1.1		0.8 ± 1.1		0.8 ± 1.1		0.8 ± 1.2	

**Table 4:** Experimental results of core–sheath configuration of PVP 60% and PEO 50%.

	PVP 60/PEO 50							
	Core	C-S	Core	C-S	Core	C-S	Core	C-S
Pressure (MPa)	0		0.1		0.2		0.3	
Speed (RPM)	12,000		12,000		11,800		11,600	
Fibre mass (g)	0.25 ± 0.01	0.33 ± 0.01	0.28 ± 0.01	0.41 ± 0.02	0.32 ± 0.02	0.49 ± 0.02	0.35 ± 0.02	0.53 ± 0.03
Production rate (g/h)	30 ± 1.5	40 ± 2.0	34 ± 1.7	50 ± 2.5	39 ± 2.0	58 ± 2.9	42 ± 2.1	64 ± 3.2
Diameter (µm)	2.8 ± 1.3	4.6 ± 2.0	2.4 ± 1.3	4.1 ± 2.0	2.2 ± 1.2	4.0 ± 2.1	2.1 ± 1.2	3.8 ± 2.1
Sheath thickness (µm)	0.9 ± 1.2		0.9 ± 1.2		0.9 ± 1.2		0.9 ± 1.2	

## Data Availability

Data are contained within the article.
